# Impact of Repetitive DNA Elements on Snake Genome Biology and Evolution

**DOI:** 10.3390/cells10071707

**Published:** 2021-07-06

**Authors:** Syed Farhan Ahmad, Worapong Singchat, Thitipong Panthum, Kornsorn Srikulnath

**Affiliations:** 1 Animal Genomics and Bioresource Research Center (AGB Research Center), Faculty of Science, Kasetsart University, 50 Ngamwongwan, Chatuchak, Bangkok 10900, Thailand; syedfarhan.a@ku.th (S.F.A.); worapong.si@ku.th (W.S.); thitipong.pa@ku.th (T.P.); 2 The International Undergraduate Program in Bioscience and Technology, Faculty of Science, Kasetsart University, 50 Ngamwongwan, Chatuchak, Bangkok 10900, Thailand; 3 Laboratory of Animal Cytogenetics and Comparative Genomics (ACCG), Department of Genetics, Faculty of Science, Kasetsart University, 50 Ngamwongwan, Chatuchak, Bangkok 10900, Thailand; 4 Special Research Unit for Wildlife Genomics (SRUWG), Department of Forest Biology, Faculty of Forestry, Kasetsart University, 50 Ngamwongwan, Chatuchak, Bangkok 10900, Thailand; 5 Amphibian Research Center, Hiroshima University, 1-3-1, Kagamiyama, Higashihiroshima 739-8526, Japan

**Keywords:** reptile, DNA repeat, genome, chromosome, transposable element

## Abstract

The distinctive biology and unique evolutionary features of snakes make them fascinating model systems to elucidate how genomes evolve and how variation at the genomic level is interlinked with phenotypic-level evolution. Similar to other eukaryotic genomes, large proportions of snake genomes contain repetitive DNA, including transposable elements (TEs) and satellite repeats. The importance of repetitive DNA and its structural and functional role in the snake genome, remain unclear. This review highlights the major types of repeats and their proportions in snake genomes, reflecting the high diversity and composition of snake repeats. We present snakes as an emerging and important model system for the study of repetitive DNA under the impact of sex and microchromosome evolution. We assemble evidence to show that certain repetitive elements in snakes are transcriptionally active and demonstrate highly dynamic lineage-specific patterns as repeat sequences. We hypothesize that particular TEs can trigger different genomic mechanisms that might contribute to driving adaptive evolution in snakes. Finally, we review emerging approaches that may be used to study the expression of repetitive elements in complex genomes, such as snakes. The specific aspects presented here will stimulate further discussion on the role of genomic repeats in shaping snake evolution.

## 1. Background

Snakes are a fascinating and unique lineage of reptiles, and comprise a rich tapestry of species (~3889 sp.) with extensive phenotypic diversity, from the loss of limbs to the development of extra-long bodies [1,2]. Snakes constitute two major recognized groups at the infraorder rank: (i) Scolecophidia, commonly termed “blind” snakes, and (ii) Alethinophidia, comprising Henophidia (pythons, boas, and other “primitive” snakes) and Caenophidia (advanced snakes) [3,4]. Diverse evolutionary adaptive changes have culminated in extreme phenotypic adaptations, such as an elongated body shape, chemical and thermal perception, loss of limbs, and venom systems, all of which distinguish advanced snakes from other squamates [1,5]. These adaptive changes in snake phenotypes have motivated researchers to study the genetic aspects with mechanistic insights into unique snake biology. Interest in snake genetics stems from the biomedical importance of snakebites in many developing countries [6], the potential for discovery of novel drugs, and the development of antivenoms [7]. Additionally, unique evolutionary features in snakes are of interest to fundamental research [3,8]. Snakes represent an enriched system for studying various extreme and unique biological features.

The molecular basis of snake-specific morphological and physiological traits remains poorly understood. A genome-level understanding of these traits could provide novel insights for vertebrate biology and medicine. Thus, snakes have emerged as useful models for studying genetic mechanisms that have undergone unique and extreme adaptations [9,10]. Despite the importance of snakes as models for basic and biomedical research, little is known about their genomes [11]. Recent rapid advances in next-generation sequencing (NGS), integrated with the development of snake chromosome maps, have provided in-depth insights into these questions by exploring snake genomes [12,13,14,15,16,17]. Genomic data have been generated for several snake species, including genome assemblies for king cobra (*Ophiophagus hannah*, Cantor, 1836) [18], Indian cobra (*Naja naja*, Linnaeus, 1758) [19], rattlesnake (*Crotalus viridis*, Rafinesque, 1818) [20], Burmese python (*Python bivittatus*, Kuhl, 1820) [21], and numerous other species [10,12,13,16] (Table 1). Most snake genomes have been generated to explore different genetic aspects, focusing on the repertoire of venom genes and evolutionary analyses [12,13,16,22]. Previous research has concentrated on the study of genes (coding regions), but repetitive DNA sequences, which constitute more than 40% of a snake’s genome, remain poorly understood [23,24,25].

Repetitive sequences are highly mutable regions, which can induce variation in genome size, structure, and function [26,27,28]. The genomic contents of repetitive sequences in eukaryotes vary from 12% in the roundworm (*Nematoda*) genome to 96% in the grasshopper (*Stauroderus scalaris*, Fischer-Waldheim, 1846) genome [29,30,31]. This fraction of the genome, which was previously considered to be junk DNA, encodes several important biological functions, and impacts on adaptation and diseases, such as tumorigenesis and neurodegenerative disorders [32,33]. Repetitive sequences are mainly classified into tandem repeats, such as satellite DNA (satDNA) [34], and interspersed repeats, including transposable elements (TEs) [35]. SatDNA repeats are fast-evolving sequences that constitute either highly repetitive, highly conserved, or both monomers in eukaryotic genomes, ranging from 150 to 400 bp in length [36]. Apart from satDNA, repetitive DNA sequences also comprise TEs that are highly polymorphic, with the ability to jump from one genomic location to another, thus contributing to genome plasticity [35,37]. Given their intense variability, repetitive sequences are further characterized as genus-, species-, population-, or even individual- and chromosome-specific elements [38,39].

Snake genomes are particularly interesting models for studying TEs, due to their unique, highly divergent repeat landscapes, and species-specific abundance of certain elements. Studies have reported the diversity of repeat contents and the highly variable structure of TEs in snakes [22,23,25,40]. Marked differences in repeat content and transcriptional activities were observed between copperhead (23-fold greater levels of TE-related transcripts) and python [23]. Snake genomes challenge the commonly accepted paradigm that genome size and repeat content are tightly linked [40], as well as the prevailing view that large variation in repeat content tends to be characteristic of major clades, rather than highly dynamic within clades. Because of this large-scale genetic variability and genomic plasticity, TEs can generate substantial genetic mutations in snake genomes, providing a source of adaptive evolution through genome diversification under natural selection. Recently, comparative and functional genomic studies have provided insights into the role of TE-mediated genome plasticity in the adaptive evolution of many organisms [41,42,43,44,45,46]. Due to their phenotypically diverse radiation, snakes have become increasingly important model systems for developmental biology, evolutionary ecology, and molecular evolution and adaptation. Snakes appear prone to the horizontal transfer of TEs (HTT) and microsatellite seeding [40,47,48]. In addition, snakes exhibit unique characteristics related to sex determination systems. Like many of its reptilian relatives, sex determination in the ancestral snake species is thought to have involved temperature and lacked the influence of sex chromosomes. For example, boa can still undergo occasional parthenogenesis and produce viable WW offspring [49], consistent with having one of the most primitive vertebrate sex chromosome pairs hitherto reported. At least three recombination suppression events occurred between Z and W in the ancestors of advanced snakes, leading to the generally degenerated W chromosome that has been observed in the five-pacer viper [22]. Thus, snakes are also suitable models for studying the mechanisms of sex chromosome evolution and elucidating how different snake lineages determine sex genetically (refer to the next section).

In snake genomes repetitive DNA has expanded at unprecedented levels; however, it is unclear how these repeats are spread across the genome [40]. One well-accepted hypothesis proposes that repeats follow cohesive/concerted evolution, leading to sequence homogenization [47]. During their evolution, variations in repeat sequences proliferate and mediate chromosomal rearrangements, resulting in either deleterious or positive effects. Consequently, repeats are interesting markers that can be used to study phylogenetic and taxonomic relationships [24,50,51,52]. The high level of specificity of repetitive sequences is an important tool for understanding evolutionary dynamics. They are also termed “tuning knobs” in the evolutionary process [53,54]. Understanding repetitive sequences allows an in-depth view of genome organization, chromosomal changes, and several other genomic phenomena such as gene expression, chromatin accessibility, and epigenetics [55,56].

Despite ongoing studies and a wealth of knowledge on repetitive sequences, a comprehensive understanding of their diversity, evolution, and functional impact remains dominated by studies on mammalian genomes [57,58,59]. Perspectives from snake genomes are particularly interesting given their unique biology, giving rise to questions on whether these repeats contribute to species-specific diversity and intense speciation. Other important research questions concern the level of genomic abundance among different snake lineages. For example, which specific elements of the snake repeatome are expressed at high or low levels? Does transcriptional activity of these repeats vary or remain consistent across lineages? What biological functions might these transcriptionally active repeats encode, and how do they impact snake genomes in biological terms? Most repetitive sequences in snakes, particularly TEs, may be silenced through epigenetic mechanisms [22]. However, certain elements may be transcribed and co-opted, and the transcripts of such repeats may interact with gene transcripts to contribute important biological functions. To support this hypothesis and to address these questions, we discuss evidence from available genomic and transcriptomic data, together with the most recent findings. We introduce snakes as model systems for studies on repetitive DNA and describe their genomic organization of sex chromosomes and microchromosomes. We detail the latest findings, highlighting different types of repetitive elements in snake genomes, and present a comparative view of snake genomes. We discuss the putative role of repeat transcripts in the adaptive evolution of snakes, and outline the contribution of snake cytogenetics to the exploration of repetitive DNA, and the recent shift toward high-scale genomics and the emergence of novel approaches to study repeat expressions.

## 2. Snake Genomes: A Model System for Studying Repetitive DNA in the Context of Chromosome Evolution Dynamics

Repetitive sequences are present at a higher abundance in distinct loci of specialized chromosomes, such as microchromosomes, supernumerary chromosomes, and sex chromosomes compared with autosomes [25,34,51]. Snakes present high diversity, especially in their sex-determining strategies, making them an interesting group for investigating the evolutionary trajectories of sex chromosomes. Different vertebrates have adopted an array of sex determination strategies [60]. However, among several non-avian reptiles, snakes display higher diversity, including temperature-dependent sex determination (TSD), genotypic sex determination (GSD), and GSD with the influence of temperature (for review see [61]). Moreover, the variant modes of sex chromosome systems including ZW, XY, and other polygenic complex determination systems can exist in snakes within the same genus, and within allopatric populations of the same species [62,63,64,65]. In addition, transitions (sex chromosome turnover) can also occur between these modes in snakes [66]. Such high dynamism highlights the complex evolutionary history of sex chromosomes in this group. In many snakes, master sex-determining genes remain unknown. Due to the high variability in sex determination modes and sex chromosomes between species, it is not surprising that the evolution of sex chromosomes in snakes remains of interest. A common feature of sex chromosomes is the accumulation of repetitive DNA, which accounts for their diversification and evolution. The heterochromatin part is highly enriched in repetitive DNA (TEs and satellites), which can differ substantially in number between sexes [30,51,67,68]. TEs can play an important role in sex chromosome differentiation [69,70,71]. Insertion of TEs near the sex-determining locus can suppress recombination by driving variations between sex chromosomes. TEs can promote ectopic recombination, facilitating genomic rearrangement to further suppress recombination [72,73].

One notable feature of snake genomes is that the sex chromosomes apparently exhibit partial dosage compensation [74], which can balance the expression of sex-linked and autosomal genes in the heterogametic sex. No mechanisms underlying partial dosage of genes or regions have been identified in snakes. In rattlesnakes, the ratio of female/male gene expression is regionally variable across the Z chromosome, suggesting incomplete dosage compensation driven by regional or gene-specific mechanisms [10]. It is assumed that a female-biased regulatory mechanism, estrogen response elements (EREs), might explain dosage-compensated regions. Evidence for ERE accumulation on the Z chromosome of the rattlesnake and the five-pacer viper indicates that EREs accumulated early in the evolution of the snake Z chromosome, providing evidence for a potential role of EREs in dosage compensation in ZW systems [10].

**Figure 1:**
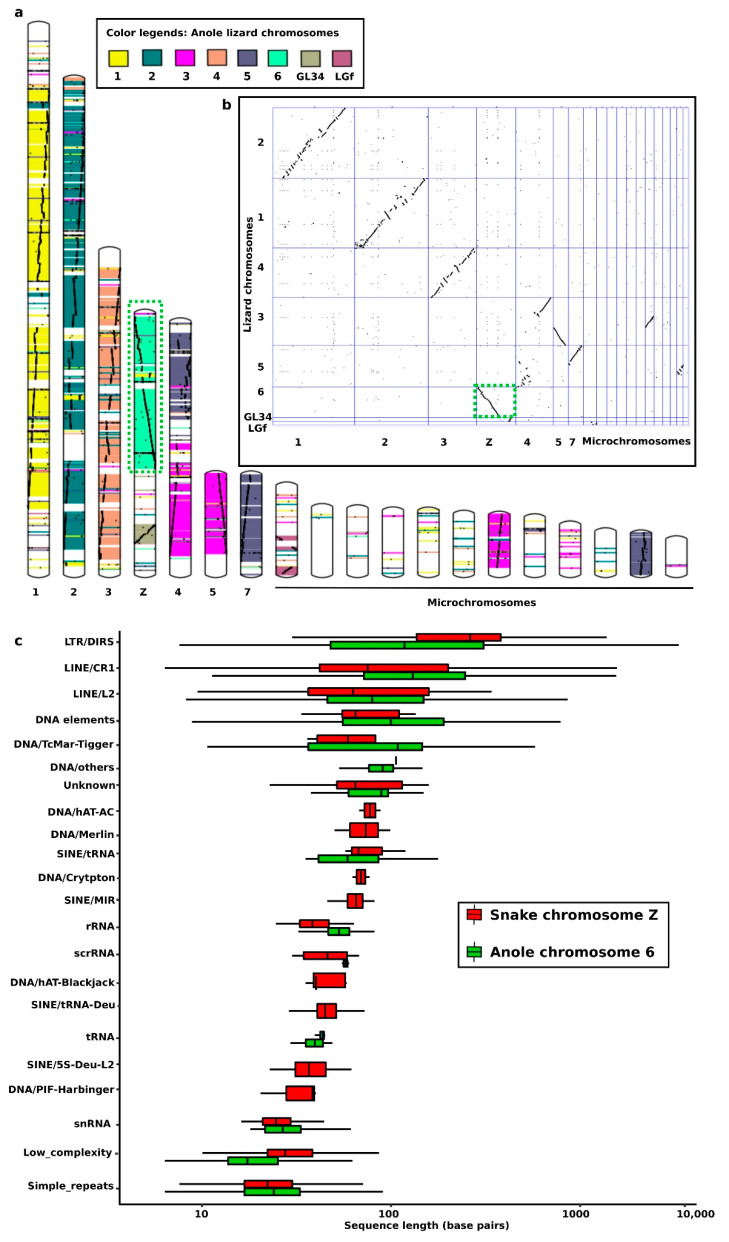
Comparative analysis of the Indian cobra genome versus anole lizard genome. (**a**) In silico chromosome painting showing cross-species homology, confirming the similarity between chromosome 6 of lizards and chromosome Z of snakes. Different colors represent distinct chromosomes. (**b**) A dot-plot view of genomic comparisons indicating different evolutionary patterns, suggesting that chromosomal rearrangements, such as inversions, have reshaped snake sex chromosome evolution. Homology between both chromosomes Z and 6 is highlighted with a dotted box. (**c**) Distribution of genomic repeats on the Z chromosome of Indian cobra showing specific expansion of repeat elements, such as CR1.

Snakes show diverse ranges of sex chromosome differentiation across disparate lineages [59,65,74,75,76,77,78]. Karyotype data indicate that most snakes have a ZZ/ZW sex chromosome system; however, heteromorphic and W chromosomes have evolved in several evolutionary phases [65,79,80,81,82]. First, an ancestral autosomal pair mutated to form a sex determination region and was transformed into homomorphic pair of proto-sex chromosomes. This was followed by heteromorphic differentiation resulting in the formation of a proto-sex chromosome with cessation of recombination and gain of male-female biased sequences. The proto-sex chromosome subsequently underwent structural changes, such as rearrangements, gene degradation, repeat accumulations, and heterochromatinization. However, certain snakes, such as python and boa, may also exhibit an XX/XY system [49]. Two major transitions, which resulted in sex chromosome turnover between these two systems, may have occurred in the Pythonidae and Boidae families [65,83]. An understanding of the genomic content can help to elucidate how significant differences in morphology and size between heteromorphic sex chromosomes emerged and contributed to turnovers. The gene content is relatively well conserved between heteromorphic sex chromosomes; however, repeat contents may contribute significantly to high variability as they are important structural and evolutionary elements [75,76,77,78,82,83]. Repeats can reshuffle sex-linked genes, and facilitate suppression of recombination while promoting chromosomal rearrangements [24,74,75,76,77,78]. Sex chromosome differentiation is linked to heterochromatin formation owing to the enrichment of TEs and satDNA in specific regions known as heterochromatic blocks [84,85]. The number of repeats can vary markedly between sexes due to the differential number of repeats on X/Y or Z/W sex chromosomes in heterogametic individuals [85,86]. In the case of snake Z and W chromosomes (the W chromosome is usually smaller than the Z chromosome), a comparative analysis of the Indian cobra (*N. naja*) genome revealed higher amplification of multiple repeats on Z chromosomes [75,76,77,78]. Similarly, genes and repeat contents of Y/W chromosomes also vary between amniote species [87]. Nevertheless, snake W chromosomes share striking cross-species homology with sex chromosomes of amniotes. Several bacterial artificial chromosome (BAC) sequences are partially homologous and have been successfully mapped on W sex chromosomes of different species, including Siamese cobra (*Naja kaouthia*, Lesson, 1831 [88], Russell’s viper (*Daboia russelii*, Shaw and Nodder, 1797 [89], and the common tiger snake (*Telescopus semiannulatus*, Smith, 1849 [90], based on hybridization signals such as repeats. These W-linked BAC repeats include large amounts of long interspersed nuclear elements (LINEs) and long terminal repeats (LTRs), which share homology with squamate reptile chromosome 2 and the chicken Z chromosome [75,76,77,78]. This multi-chromosome homology across diverse species suggests that snake sex chromosomes evolved as a result of repeat-mediated rearrangements [25,78], which drove genomic changes, such as chromosomal-scale variation and gene reshuffling, resulting in species-specific evolution and diversification at the individual or population level [27,28,91]. Different types of inter- and intra-chromosomal rearrangements, facilitated by TE insertions and repeat variations, comprised non-homologous recombination, deletion, inversion, duplication, and translocation, which further led to substantial structural reorganization of chromosomes through neocentromere and centromere repositioning [92,93]. Although these events are not completely understood, advances in snake comparative genomics may allow further insights to be gained. Recently, comparative analyses of five viper species suggested that snake genomes have a distinctive genomic architecture shaped by lineage-level expansion of respective TEs [22]. Previously, snakes were shown to share extensive inter-chromosomal homology with lizards [61,81,94,95,96,97,98]. For example, colubroid Z chromosomes are homologous to *Anolis* chromosome 6 [74] (Figure 1). Considering previous cytogenetics-based results, we compared *N. naja* and *Anolis* chromosome-scale genomes. The results showed that snake sex chromosomes share 13 of the longest homologous synteny blocks with *Anolis* chromosome 6. Whole-genome comparisons traced different evolutionary trajectories (Figure 1). Conserved linkage homologous regions suggest that snake sex chromosomes gained their genomic contents from ancestral autosomal segments, and that chromosomal loci were reshuffled through the transposition and accumulation of repeats [75,76,77,78]. Collinearity is not exclusive to Z chromosomes and interautosomal syntenies are also observed. Additionally, we performed a comparative analysis of TE families between snake Z chromosome and anole chromosome 6. This analysis assessed whether sex chromosomes have a higher tendency of accumulating certain types of repetitive DNA compared to anole chromosome 6 (Figure 1c). We found that certain elements in the snake genome may be specifically expanded at higher levels on chromosome Z, such as DNA/hAT-Ac, DNA/Crypton, DNA Merlin, SINE/MIR, SINE-Trna-Deu, DNA/Hat-BlackJack, scRNA and DNA/PIF-Harbinger, in contrast to chromosome 6.

In addition to sex chromosomes, snakes possess microchromosomes, which are also found in avian and many reptilian karyotypes, except for the crocodilian lineage [94,95,96,97,98,99,100,101,102,103,104,105,106,107,108,109]. Microchromosomes are hypothesized to have emerged as evolutionary by-products of fission events in ancestral amniote macrochromosomes, followed by centromere inactivation leading to karyotypic diversity among snake species [61,94,95,96,97,98]. In certain reptilians, such as crocodile or gecko lineages, these microchromosomes may have disappeared as a result of fusion with macrochromosomes [98]. The genome biology of microchromosomes is intriguing due to their unique features, such as enriched GC contents (around 48% on average) and higher gene density compared with that of macrochromosomes [110,111]. However, a comprehensive comparative analysis of repeat density between micro- and macrochromosomes is lacking in snakes. Cytogenetic mapping has shown that microchromosomes of snakes have a preferential tendency to gain telomeric repeats, as reported in iguanian lizards and birds [75,94,112,113,114,115]. An abundance of telomeric and other repeats may impact the differences in recombination rate between micro- and macrochromosomes. The microchromosomes of chicken show a high frequency of recombination [116,117,118]. Microchromosome-specific amplification of satellite repeats has been observed in certain reptiles, such as turtles, but not in snakes [103,119,120]. It remains unclear whether genomic compartmentalization into micro- and macrochromosomes increases the rate of evolution to drive species diversity. Species-rich snake genomes harbor both micro- and macrochromosomes; however, members of Crocodylia, which have a lower species richness, rarely exhibit genome rearrangements, suggesting that the ancestral crocodilian karyotype was highly conserved with no microchromosomes [98,103]. It remains unclear whether repeat-enriched regions induce genomic compartmentalization, and what role they played in the evolutionary success of microchromosomes. Comparative repeatomic landscapes and chromosomic analyses using snakes as model systems are key to elucidating the enigmas of sex chromosomes and microchromosomes.

## 3. Repeat Abundance in Snake Genomes

**Figure 2:**
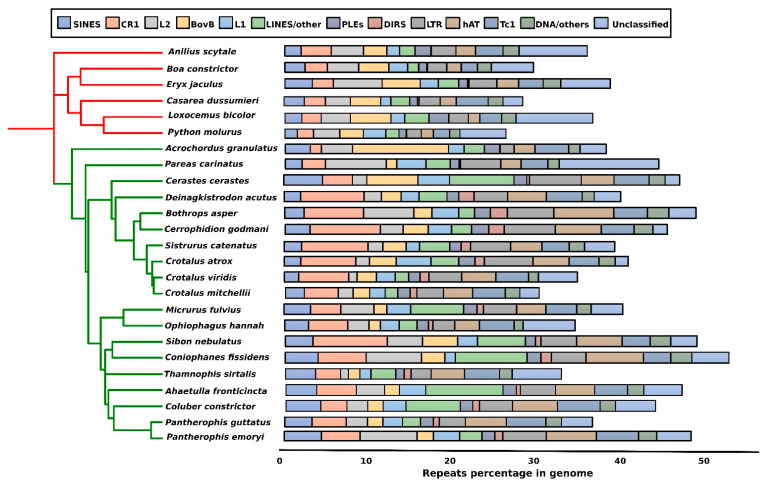
Genomic comparison of repetitive elements in advanced and primitive snake species. The phylogenetic tree was derived from TimeTree by Kumar et al. (2017) [130] and customized with iTOL v6 (https://itol.embl.de/, accessed on 9 April 2021). The genomic percentage for each snake species genome is shown as a bar chart. The red and green branching of the tree represents the species of primitive and advanced snakes, respectively.

Genome size varies between species, with most snake genomes ranging from 1.3 to 3.8 Gbp [25,40,121]. Such variation in genome size among species is associated with different proportions of repeats [26,122]. Snake genomes possess high variability of repeat elements, ranging from 25 to 73%, while the genomic composition varies among species (Figure 2). This relatively high degree of genomic repeat variation within a short evolutionary time scale in snakes, contrasts with that in birds and mammals. Unlike the lineage-specific patterns observed in mammalian genomes, the variation in repetitive content in snakes may arise even between species and within the same genus, e.g., within the genera *Ophisaurus* (44.8–48.9%), *Coniophanes* (59.4–73%), and *Crotalus* (35.3–47.3%) [40]. In pythons, the content of identifiable repeat elements is low (21%), similar to that in bird genomes (around 10–20%), whereas the content in the copperhead is higher (45%), and comparable to that in mammalian genomes (more than 40%) [123]. Greater repeat content in the copperhead arises from the recent expansion of different microsatellites and TE families, e.g., the abundance of TEs in the copperhead genome is 23-fold higher than that in the python [40]. Certain families of TEs, such as LINEs-like elements, have experienced high levels of expansion in several species of advanced snakes, such as *Cerastes cerastes*, *Coniophanes fissidens*, *Micrurus fulvius*, *Sibon nebulatus*, *Ahaetulla fronticincta*, and *Coluber constrictor*. All six primitive snake species compared (Figure 2) were found to display a low abundance of CR1 and LTRs, which were higher than those in many species (*Deinagkistrodon acutus*, *Bothrops asper*, *Cerrophidion godmani*, *Sistrurus catenatus*, *Crotalus atrox*, *C. viridis*) of the advanced snake lineage. In addition, BovB is found at high abundance in the genome of the marine file snake (Figure 2) (*Acrochordus granulatus*), which is indicative of HTT events as also reported by Galbraith et al. [48] in several species of sea snake. This variability can contribute to genome diversification between primitive and advanced snakes, and even to variation in species-specific elements, thereby reshaping homomorphic or heterophonic sex chromosomes and karyotypic variability with different numbers of microchromosomes.

Among reptiles, genomic repeat comparisons (snakes versus other non-avian reptilian lineages) provide interesting insights into reptile diversification and speciation [25]. For example, the anole lizard has a well-characterized genome, which can be distinguished from that of snakes by different aspects of repetitive DNA, such as their abundance and evolutionary age. For example, the average genomic percentage of Tc1-Mariner is 2.4-times more abundant in colubroid snakes (4.23%) than in lizards (1.7%) [40,124]. Kimura divergence analysis of repeat landscaping showed that TEs in snakes were older compared with those of anole lizard TEs [25,123]. Nevertheless, some young elements have also experienced recent expansion in snake genomes, e.g., snake1, CR1, LINEs, and BovB [40,78,125]. The CR1 element has also expanded in the crocodilian lineage, constituting around 10% of the total genome. Both ancient relics and recent activity of this retroelement have been detected in different reptiles [126]. Interestingly, birds and lepidosaurs retained the fewest ancient CR1 lineages among amniotes [27,127]. The accumulation of variable CR1 copies has been reported in vipers, boas, and pythons [22]. CR1 has further diverged into subfamilies, with a recent genomic expansion of these elements reported in the turtle lineage [128,129]. Apart from CR1, other transposons that dominated snake genomes have included hAT-Charlie, Tc1/Mariner, Gypsy, and L2 [40,123] (Figure 2).

Findings based on DNA reassociation indicate that snake species exhibit substantial variation in repeatomic contents, mainly between species of pythons and colubroids [131,132]. Recent advances in genome sequencing technologies have facilitated the thorough investigation of snake genomes, and provided key insights into how repeats reshape evolution and contribute to differences in genome size [121,133]. Transposons are a major component of eukaryotic repeats. These can be divided into two classes and snake genomes containing all three groups, including Class I, containing LINEs and LTR retrotransposons, and Class II, containing DNA transposons [134]. In particular, advanced snake repeatomes are more diverse than those of primitive snakes, and present notably higher expansion of TEs [40]. Interestingly, advanced snakes also present substantial variability of GC contents in their genomes. It remains unclear whether shifts in GC content are related to genomic repeat landscapes in colubroid snakes. Specific TEs that underwent a significant increase in copy number in advanced snakes include CR1s, RTE-BovBs, Rex1, and L2s (Figure 2). These TEs are important phylogenetic markers in studies investigating the diversity of snake lineages. Recent exploration of a retroelement termed Bov-B LINE shed light on the diversity of snake lineages [125]. Sequence variability of Bov-B LINE resolved the complex phylogenetic framework among eight diverse snake lineages, including Henophidian (Cylindrophidae, Boidae, and Pythonidae) and Caenophidian (Acrochordidae, Viperidae, Homalopsidae, Elapidae, and Colubridae) snakes. Further analysis indicated that this element might have invaded the snake genome through horizontal gene transfer (HGT). The Bov-B LINE might have been transferred by tics from squamates to bovids [135]. Similar cases of transposon-mediated HGT have been observed in non-snake lineages, such as nematodes, teleosts, and birds [136,137]. These HGT events induced structural changes in the genome, which can drive adaptative evolution, such as transitional changes from marine to terrestrial environments [138,139]. Recent evidence acquired from genome analyses of the olive sea snake (*Aipysurus laevis*, Lacépède, 1804) [140] identified HGT linked with six novel retrotransposons in sea snakes following their marine transition within the last 18 million years [141]. Interestingly, these novel elements are absent in terrestrial animal genomes, which presented high similarity to retrotransposons present in fish, corals, and marine sea kraits. Moreover, these sea snake-specific retrotransposons are likely to be expressed and highly abundant in the *A. laevis* genome.

In some instances, TEs are embedded inside gene clusters of snake genomes. One example is the Hox family, which includes developmental genes that play a crucial role in the control of embryogenesis [142]. The genome of the corn snake (*Pantherophis guttatus*, Linnaeus, 1766) [142] specifically accumulated TEs within the intronic and intergenic regions of Hox clusters, thus contributing to the expansion of gene size [143]. In addition to TEs, snake genomes harbor a higher abundance of repeat arrays of microsatellites than all eukaryotic genomes reported to date [24,25,40]. Repeat annotations of colubrid snakes (e.g., *Coniophanes fissidens*, Günther, 1858) [144] have estimated microsatellite levels at 14% [40]. Compared to other vertebrates (fish and mammals) with similar microsatellite repeats, snakes tend to present lineage-specific variation in genomic abundance of these microsatellites, even on a species level. This is evident from the repeatomic density analysis (number of loci/Mbp) of microsatellites, with differences ranging from 10.9- to 16.6-fold, with primitive snakes presenting the lowest, and advanced snakes presenting the highest density of these repeats. Extreme variability of microsatellite genomic contents in snakes has set a new benchmark, exceeding the high diversity of these repeats previously recorded in fish genomes [145,146]. By contrast, mammalian and avian genomes tend to possess the lowest variation in microsatellite density (1.8-fold loci/Mbp and 2.8 bp/M). An interesting aspect of microsatellite evolution in squamate genomes is the abundance of specific motifs (4-mer ATAG and 5-mer AATAG) with extreme degrees of expansion in advanced snakes. It remains unclear how advanced snake genomes accumulated such high abundance of these microsatellite motifs. Previous studies have shown that a specific mechanism, known as “microsatellite seeding”, can lead to extreme levels of microsatellite genomic expansion [25,40]. This process of microsatellite seeding in colubroid snakes is driven by the expansion of CR1-L3 LINEs, as indicated by the high genomic representation of these transposons located within regions adjacent to microsatellites. Tandem repeat seeding is also enriched in the surrounding regions of highly duplicated venom genes in snakes [22,147]. Further investigation is necessary to determine whether TE-satellite hybrid repeats impacted the evolution of prominent phenotypes in snakes (i.e., venom evolution).

## 4. Snake Repeats in the Cytogenetics Era

Cytogenetic studies of snakes have revealed intriguing findings in the chromosome-specific mapping of repeats using fine-scale molecular cytogenetic techniques [61,75,76,77,78,81]. The landmark Ohno hypothesis, based on snake comparative cytogenetics, proposed that sex chromosomes originated from autosomes [79]. Cytogenetic analysis is also an effective tool for understanding the highly repetitive nature of centromeric heterochromatin to investigate genomic compartmentalization between macro- and microchromosomes in snakes. Techniques such as C and G banding have shown that microchromosomes are heterochromatic and indicative of repeat enrichment [148]. Initially, cytogenetic studies were based on radioactive labeling of the repetitive sequences (rRNA and satDNA), whereas subsequent research utilized fluorescence in situ hybridization (FISH) to map these sequences on chromosomes [149]. Different types of DNA vectors, such as BACs, cosmids, and fosmids, have been used as DNA libraries carrying large probe sequences (30–300 kb). These approaches revolutionized the field of cytogenetics during the 1990s, allowing researchers to perform chromosome painting and multi-color hybridization analyses [150].

FISH is a successful cytogenetic method [149], and has been applied extensively to map repetitive regions, most commonly rDNA sequence probes. The first case of rDNA FISH mapping in snakes was accomplished on macrochromosomes in Viperidae, revealing a translocation that involved the repetitive region from micro- to macrochromosomes [151]. Conversely, the rDNA cluster may also remain highly conserved at the subspecies level, as revealed by Southern blotting analysis in neotropical snakes [152]. Nucleolar organizer regions, in which rDNA is particularly clustered, have been observed with similar organization on the microchromosomes of snake species, including *Liophis poecilogyrus shotti* [153], *Bothrops jararacussu*, Lacerda, 1884 [154], and the South American rattlesnake (*C. durissus terrificus*, Linnaeus, 1758 [19,155]. Likewise, in the viperid *C. viridis* Rafinesque, 1818 [20] and the colubrid *Masticophis flagellum* (Shaw, 1802) [156], rDNA repeats are commonly present on two pairs of microchromosomes [157]. Snake cytogenetics have also provided insights into the chromosomal distribution of various satellite repeats, which can be locus-specific. For example, the PBI-DdeI satDNA is clustered within centromeric regions. Mapping of PBI-DdeI satDNA recorded chromosome W-specific hybridization signals in Siamese cobras (*Naja kaouthia*, Lesson, 1831 [88], which was indicative of its female-specific amplification [24]. A cytogenetic-based study of Amazonian puffing snakes of the genus *Spilotes* mapped five microsatellites to chromosomes [82]. Differential patterns of hybridization signals were observed for different species of *Spilotes*, with intense clusters of specific (AC)_15_ monomers on the W chromosome. (AG)_15_ repeat units were found on the centromeres and telomeres of the first and fourth autosomal pairs, and on the W chromosome in *Spilotes sulphureus*, Wagler, 1824 [82,158], whereas in *S. pullatus* (Linnaeus, 1758) [19] bitelomeric markings of (AG)_15_ were detected on all chromosomes, with intense accumulation within the centromeric position of the second pair and on the long arms of the W sex chromosome. Other microsatellites, including (ATCC)_8_, (ATTC)_8_, and (GATA)_8_ repeats, show W chromosome-specific signals with distinctive patterns for each analyzed species. Cross-species BAC hybridization is a promising cytogenetic approach that can produce a detailed chromosome map of repetitive elements and has been applied to many snake species [75,76]. A chromosome map of Siamese cobras (*N. kaouthia*) was constructed using a range of BACs derived from chicken and zebra finch libraries using FISH. The BAC comparisons revealed that the Siamese cobra shares a high number of syntenic regions between chromosomes 2 and Z, and BAC annotation revealed different types of repetitive elements, particularly TEs. FISH mapping has shown chromosome W-specific enrichment of telomeric and microsatellite repeats in cobra and other squamates [24,75]. Molecular cloning and cytogenetic techniques have also found an abundance of satellite repeats in heterochromatic regions, such as centromeres. For example, centromeric regions of habu snakes (*Protobothrops flavoviridis*, Hallowell, 1861) [159] and Burmese pythons (*P. bivittatus*, Kuhl, 1820) [21] harbor three different families of satDNA, specifically 168 bp PFL-MspI,196 bp PBI-DdeI, and 174 bp PBI-MspI [160]. In elapid snakes, such as banded kraits (*Bungarus fasciatus*, Schneider, 1801) [161], species-specific repeats have been mapped exclusively on the short arms of the W chromosome [162]. Cross-species hybridization of DNA clones and satellite repeat mapping on snake chromosomes have provided evidence for evolutionary conservation among diverse taxa. For example, cloning of 2.5 kb satellite sequences from *Elaphe radiata* (Pope, 1929) revealed the common occurrence of these sequences in fly and mouse genomes; however, organization on a chromosomal level differed among species [163,164]. Similarly, comparative genomic hybridization of repetitive DNA between birds and snakes revealed interesting molecular aspects of W sex chromosome degeneration [75,78,165]. In addition, W-specific repetitive regions (comprising *Bkm* and 18S rDNA repeats) have been identified in representative species of Australian snakes, including the water python (*Liasis fuscus*, Peters, 1873) [166] and the Bird’s Head Peninsula groundsnake (*Stegonotus cucullatus*, Duméril, Bibron, and Duméril, 1854) [167,168]. Cross-species FISH mapping and molecular cloning are still performed in snakes; however, the emergence of NGS technologies has signaled a shift from cytogenetics to cytogenomics [25]. Recent progress in snake genome sequencing suggests that snake cytogenetics may soon become obsolete, whereas genomic tools can resolve most biological questions pertaining to repeat evolutionary dynamics. Despite the latest developments in genomic techniques, important findings can still be obtained from fundamental cytogenetic-based repeat mapping on chromosomes. This includes the evaluation of chromosome number and morphology, which provide basic data for the advanced molecular and bioinformatic analyses of repeat identification. In addition, a combination of genome sequencing and physical chromosome mapping can be a highly productive approach, particularly for anchoring assembled scaffolds onto chromosomes. The integration of cytogenetics and chromosome-level genome assembly has emerged as a new field, termed “chromosomics” [169], which can be considered a “golden path” to an improved understanding of repeatomes in snakes.

## 5. Transcriptomic Profiling and Expression Dynamics of Principal Repeats in Snakes

**Figure 3:**
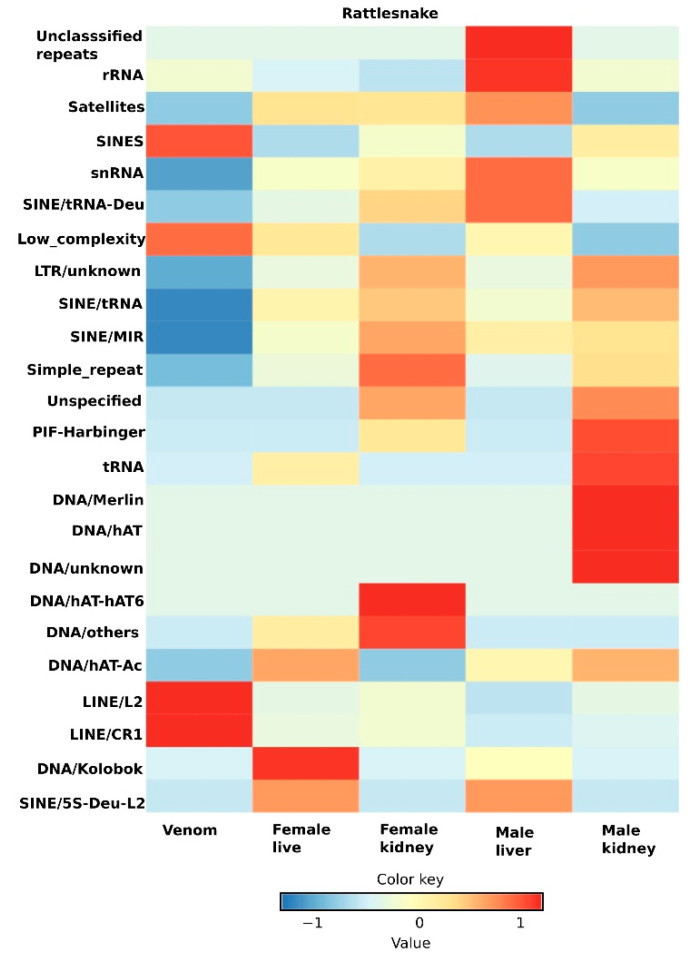
Heatmap illustrating differential expression data of genomic repeats in rattlesnakes. Comparisons are shown for different tissue samples and repeat families on the x-axis and y-axis, respectively. Log fold-change (log FC) values denote differential expression of repeats in different tissues. The transcriptional activity of repeats was compared between tissue sample groups using the approach described in Supplementary Note S1.

In the post-genomics era, NGS technologies have enabled researchers to investigate the complex nature of repetitive elements and decipher their functional impacts. Repeats that were initially considered to be junk or parasite DNA and were routinely removed or overlooked in genomic analyses, are now known to be actively transcribed and encoded, with several important biological functions. Repeat transcripts have been identified in numerous model and non-model organisms [38,170]. In snakes, most attention has focused on understanding the genomic-level organization of repetitive elements (using cytogenetic and genomic tools as described in “Section 7”). Despite extensive progress, transcriptional activity is still not well studied. Yin et al. (2016) examined the expression of repeats in the five-pacer viper (*Deinagkistrodon acutus*, Günther, 1888) [171] using transcriptome sequencing in several tissues, including the brain, liver, venom, ovary, and testes [22]. The viper genome is reported to possess the highest percentage abundance of TEs (47.47%) of any snake genome sequenced to date. In a rare study on snake repeat transcriptomics, Yin et al., 2016, reported that most TEs in the viper genome were silenced, and that few elements were differentially expressed in all analyzed tissues. Interestingly, in the brain, almost all major types of TEs were transcribed [22]. These results prompted researchers to test whether TEs are assigned a role in regulating gene networks in the brain. Notably, the genes adjacent to these highly expressed TEs were found to encode proteins with important cellular functions, including significantly enriched gene ontologies, such as environmental response and brain signaling. Although interesting insights were gained from this research, no subsequent study has evaluated or validated the transcriptional activity of repeat elements in other snake species. To assess whether genomic repetitive element transcriptional activity for different snake species adheres to the patterns observed in viper, we analyzed transcriptomic data in different tissues available in the NCBI Sequence Read Archive (Table S1, Figure S1 and Supplementary Note S1) for rattlesnake and Indian cobra. Contrary to findings from the aforementioned study that revealed brain-specific repeats in the brains of vipers, we observed a dynamic tissue-specific pattern of transcriptional expression for each repeat type in both rattlesnakes and Indian cobras. The dynamism of TE transcriptional activity has also been observed in copperheads (*Agkistrodon contortrix*, Linnaeus, 1766) [142] and Burmese pythons (*P. bivittatus*) [23]. In a transcriptomic-based study of liver tissues, the expression of active TEs was 23-fold higher in copperheads compared with python snakes. These elements included LINEs, CR1, and Bov-B. Interestingly, we observed venom-specific expression of low complexity repeats and retrotransposons, such as LINE/L2, LINE/CR1, and short interspersed nuclear elements (SINEs), in rattlesnake (Figure 3). These elements were silenced in the venom of Indian cobras, in which there was significant expression of satellite repeats (Figure S2). This lineage-specific transcription of distinct repeat elements suggests that repeats play important roles that may determine the unique expression of genes in snake venom. Evidence from previous reports on snakes suggested that TE activity was crucial in the evolution of major adaptations in snakes and may also include the evolution of venom repertoires [23,133,148]. Fujimi et al. (2002) reported the insertion of CR1 fragments proximal to phospholipase A2 venom genes in vipers, which might result in non-allelic homologous recombination, leading to duplication of these genes. Apart from venom-biased expression of retrotransposons (in rattlesnake) and satellites (in Indian cobra), certain repeat families (DNA/haT and DNA/Merlin) were highly active in males, but not in females [172]. Moreover, most repetitive elements in Indian cobra are silent, and very few, such as tandem repeats, are active only in the brain. We also observed transcriptional evidence of higher active expression in the livers of male rattlesnakes compared with other tissues. It is unclear what these unclassified elements represent; however, we consider that most of these elements might be SINEs, due to the similarity of their sequence length. Our analysis provides preliminary insights into the transcriptional dynamics of repetitive elements in snakes to stimulate further research into the transcriptional status of repeats in snake genomes. Future studies should include experimental validation showing how these repeats evolve to influence gene expression in venom and other tissues. Further understanding can be gained by highlighting the potential impact of repeat contents in reshaping the evolution of the snake genome.

## 6. Putative Impact of Transposable Element Transcripts on the Evolutionary Dynamics of Snakes

The impact of transcriptional and post-transcriptional processes on genomic repeats has been well documented in diverse eukaryotic taxa [38,173]. However, it remains unclear how these repeats influence snake genomes, and studies assessing their expression in reptilian lineages are lacking. Repeat transcripts, particularly TEs, play crucial roles in the regulation of gene expression through cis and trans mechanisms to drive different evolutionary processes [174]. A recent study analyzed the effect of species-specific TE transcripts on gene expression in six representative amniote genomes (human, opossum, platypus, anole, bearded dragon, and chicken), based on data obtained from RNA-sequencing analysis [175]. The magnitude of gene expression associated with TEs varied in each genome, and species-specific patterns of TE expression associated with gene expression were also observed, supporting a putative role for TEs in amniote speciation. The TE transcriptional profiles in lizard and bearded dragon revealed an association with the evolutionary age of certain elements. For example, new LINEs downregulated gene expression, whereas older LINEs upregulated gene expression. Strong correlations have been reported between the transcriptional activity of repeats and genes in various eukaryotic genomes [176]; however, how these repeats coordinate with gene expression is not well understood.

One hypothesis suggests that genomic repeats serve as functional domains of non-coding RNAs (ncRNAs) that are essential in the control of many cellular processes [177,178]. Common small ncRNAs are microRNAs (miRNAs) that regulate gene expression by influencing mRNA stability and translation [179,180,181]. In snakes, transcriptional miRNAs in juvenile and adult yellow-bellied sea snakes (*Hydrophis platurus*, Linnaeus, 1766) [142] from Costa Rica act as repressors and enhancers to regulate the mRNA translation of venom toxins [182]. In the king cobra genome, miR-375 miRNA is expressed in venom glands and regulates core gene networks that are important in the evolution of snake venom glands [13]. Future studies should focus on the origin and evolution of venom-associated miRNAs in snakes. Several studies have shown that repetitive elements produce miRNA sequences and are comprised of paralogous species-specific miRNAs [183,184,185,186,187]. For example, the genome of saltwater crocodile harbors 44% of the expressed miRNAs that are localized near the TE loci [188]. An additional type of ncRNA, termed long non-coding RNAs (lncRNAs), can bind DNA proteins and affect gene transcription [189]. An association between lncRNAs and repetitive elements, particularly TEs, has been reported. The repeat insertion domains of lncRNAs (RIDL) hypothesis states that TEs can be inserted within lncRNAs, providing a source of functional domains for the transcripts [190].

**Figure 4:**
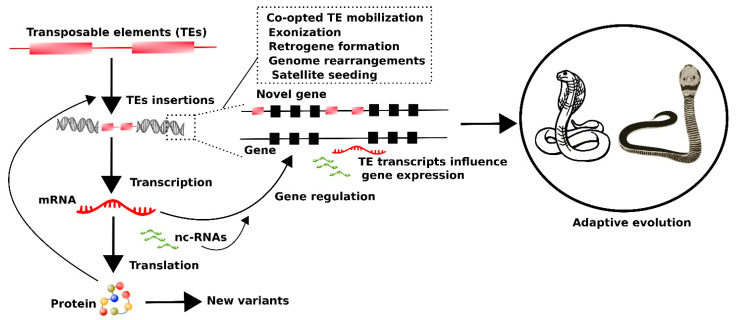
Hypothetical model of transposable elements (TEs) showing their possible impact on snake adaptive evolution. TEs insert themselves into different genomic loci and are transcribed into TE mRNAs. Some TEs are translated into proteins that mediate TE mobilization into new loci and contribute to the formation of new phenotypic variants. Other TE-derived transcripts act as non-coding RNAs (nc-RNAs) that influence the expression of genes and ultimately produce phenotypic-level variation leading to adaptive evolution. Different mechanisms are triggered by TEs, which drive this process.

A notable aspect of genomic repeat expression is their role in regulating adaptative evolutionary phenotypes. The evolution of these elements is mainly driven and constrained by their environment [191,192]. Thus, iconic adaptive evolutionary episodes for snakes might have been influenced by the expression activity of repetitive elements (Figure 4). A common perception is that TEs have a negative impact on genome integrity and host fitness [193,194,195]. However, emerging evidence suggests that TEs play a beneficial role and are crucial in driving several important genetic innovations associated with adaptive evolution, such as gene regulatory networks [196].

Growing literature concerning the genomic dynamics of TEs continues to attract the interest of evolutionary biologists on the role of TEs in adaptive evolution. TEs are mutagenic factors that can induce genetic variations in response to environmental changes in response to stress [42,192]. Thus, increased TE activity may drive genetic diversification within natural populations, enabling the emergence and subsequent selection and fixation of novel adaptive variants through natural selection [197,198,199]. Genetic diversification in fast-evolving regions is driven by structural variation through either aberrant recombination, transposition, or in combination, and repeat-induced point mutations [28,200,201,202]. The role of TEs in adaptive evolution has been elucidated through the two-speed genome concept of evolution [203], which highlights that genome evolution can act differently at repetitive and non-repetitive regions. The non-repetitive region evolves slowly, lacks repeat elements, is gene-rich, and contains core genes responsible for basic physiology. The repetitive region evolves rapidly, with abundant TEs and poor gene content, and may drive adaptive evolution.

In snakes, fundamental questions pertaining to the adaptive mechanisms of axial patterning and limb development, and venom evolution are being investigated [133,204]. Recently, knowledge into these questions has advanced based on genome and transcriptome sequencing of many snake species. Improvements in the quality of genome assembly have provided opportunities for scrutinizing the lineage-specific enrichment of repeats. Repetitive enriched regions containing TEs can trigger changes in gene function, which can drive evolution via selection for particular phenotypes [205]. Previous analyses of snake genomes and transcriptomes have detected signatures of adaptive evolution of toxin families and developmental genes in advanced snakes [9,12,206]. Elapid genomes are thought to have accumulated TEs due to environmental stress, and episodic transpositions generated inheritable genetic variation in these genes across different species, thus facilitating the evolution of adapted phenotypes. Recently, a correlation between TEs and speciation/diversification has been suggested for several mammalian lineages [207]. Empirical evidence implicates TEs in the adaptive radiation of primates [51,208], bats [209,210], and *Anolis* lizards [211]. For example, following a burst of activity in *Anolis*, TEs populated the vicinity of Hox gene clusters, the genomic regions associated with morphological adaptations to different habitats in these species. Additionally, the tuatara (*Sphenodon punctatus*) lineage, which diverged from snakes and lizards around 250 million years ago, has undergone intense expansions of TEs including recent episodes of LINE and SINE retrotranspositions. The unusual composition, abundance, and diversity of TEs, and the prevalence of recent retrotranspositions and segmental duplication in the tuatara genome, may indicate that TEs mediated diversification in reptiles [56]. Together, these findings raise the question of whether environmental-induced TE transposition can drive diversifying evolution in snakes.

Different mechanisms have been proposed as TE-mediated driving forces that contribute to the dynamics of adaptive evolution (Figure 4). TEs can encode proteins that are essential for their mobilization, and generate novel phenotypic variants [212,213]. Insertion of TEs in the intronic region of a gene can result in the emergence of a new exon, in a process known as exonization [214,215,216]. Transposition within flanking regions of the genes can also alter transcriptional expression [217]. Specific elements, such as LINE1 with retrotransposition ability, can interrupt gene transcripts [218,219]. The occurrence of TE paralogs in different genomic regions can mediate aberrant transposition [220,221] and ectopic recombination [222,223,224,225], leading to novel structural rearrangements and genomic plasticity. Adaptive traits in snakes may have been driven by lineage-specific transcriptional activity (Figure 3). For example, the transcriptional activity of LINEs in amphibious elapids provides evidence that these elements may drive ecological adaptation in marine environments. Interestingly, active LINEs are exclusively present in sea snakes and are localized in the vicinity of “ADCY4”, a gene involved in circadian entrainment [48]. Several other genes involved in the functions of adaptive features (metabolism, development, limb loss, trunk elongation, and skeletal changes) have been detected with rapid and extremely variable transcriptional responses in multiple organ systems under different environments [12,13,22,226]. Such responses involve large-scale changes in gene expression that are tightly coordinated with rapid adaptive changes in organ size and metabolism after feeding in pythons [12]. These findings with regards TE transcripts and their possible role in regulating genes linked with adaptative evolution are most intriguing, and this aspect of snake biology requires further investigation.

## 7. Measuring the Expression of Repeat Elements: Modern Approaches and Challenges

Measuring the transcription of repetitive elements has been challenging due to their low expression level and the lack of tools adapted to their unique features. Several molecular and computational tools have been developed to determine the expression of repetitive elements; however, care must be taken to ensure that active repeats are detected [227]. The most common biological aspects include investigation of transposon expression, their functional impact on genes, and other biologically active molecules, such as ncRNAs, which originate from TEs [228]. Conventional approaches for assessing the expression of repeat sequences include quantitative reverse transcription PCR, Northern blotting, and in situ RNA hybridization [229,230,231]. These techniques have several major limitations, such as the difficulty designing RNA probes and the inability to amplify mutant and defective transcript copies [227]. TE-encoded proteins can also be detected via Western blotting and immunofluorescence as complementary experimental approaches. Some of these techniques have been used to explore genomic aspects of amniotes and could also be applied to explore snake repeat biology.

A common approach is to take a genome-wide view of repeat expression using RNA sequencing [232,233,234,235]. This approach is highly effective and utilizes an increasing number of bioinformatic tools (Table 2) dedicated to the annotation of repeats and to the examination of their differential transcription. Most of these software programs implement commonly used read aligners, such as Bowtie2 [235], STAR [236], TopHat [237], and BWA [238]. These computational tools differ in their alignment algorithms, their level of resolution to classify repeats into families and subfamilies, their strategy to detect polymorphisms, and their ability to quantify chimeric transcripts [227,239,240]. One challenge related to the use of these tools is that the short reads originating from the repeats can also map to other genomic positions. These reads are termed “multimappers” and can induce bias and errors, leading to the overestimation of transcript counts. One solution to this problem of mappability is to align reads against reference genomes, retaining only the uniquely mapped reads as “unimappers” [228]. We applied a similar stringent mapping criterion during transcriptomic profiling of rattlesnake and Indian cobra repeats by adapting ‘—very-sensitive’ in the ‘—end-to-end mode’ of the Bowtie2 short reads aligner (Supplementary Note S1). This strategy can be effective, and produces fair results on repeat transcript expression; however, transcript counts of evolutionarily young TEs (with variants) might be underestimated or eliminated. Multimapping can also be challenging for the accurate estimation of transcripts for older repeat families owing to their divergence from the consensus sequence. Therefore, the interpretation of repetitive element transcription in snake genomes can be complex and difficult.

Given the extreme variability and sequence divergence of genomic repeat contents between snakes [40], it is essential to optimize approaches for the accurate and precise detection of repeats. Paired-end short-read sequencing and long-read sequencing with less relaxed alignment stringency to tolerate more mismatches are recommended to overcome this problem. Development of novel pipelines with appropriate parameters specific for studying snake repeats, integrated with experimental validation, can address the analytical complexities of identification and understanding repeats. Action is also recommended for accurate genomic and transcriptomic landscaping of repeats in eukaryotes. Lanciano and Cristofari (2020) reviewed genome-wide expression assays and presented a complete picture of TE transcriptomic profiling with challenging features [227]. For computational approaches applied to the detection of TEs, interested readers are referred to the review by Goerner-Potvin and Bourque (2018) [241].

## 8. Conclusions

The increasing availability of genomic resources and the establishment of computational tools have enabled the production of high-quality snake genome assemblies. Snake research has now fully migrated from cytogenetics to the genomic era. This shift has advanced our understanding of repeat content, which are highly diverse and abundant in snake genomes [25,40,126]. This diversity of repeat structures and composition suggests that snakes possess unique repeatomic landscape features compared with other amniotes. Unique characteristics of snake genomes include microsatellite seeding by LINEs in some species, resulting in the highest microsatellite expansion of any known amniote genome. Transcriptomic studies provide evidence that, among snake repeats, most TEs and some tandem repeats are active, and are dynamically differentially expressed in both a lineage- and tissue-specific manner. These findings imply that transcripts of snake repeats influence gene regulatory pathways and might be linked to extreme phenotypes in snakes. Detection of repeats and their transcripts is highly challenging owing to their repetitive nature, mappability, polymorphisms, and transcript identity. The recent emergence of long-read sequencing and diverse bioinformatic tools have proved highly effective for advancing repeat analysis. We anticipate that these approaches, integrated with experimental advances, will help to fill knowledge gaps concerning snake repeats and will help researchers to understand the impact of TEs and repeats on snake adaptive evolution.

## Figures and Tables

**Table 1:** Snake species for which a complete genome assembly has been published.

Species	Common Name	Assembly ID	Assembly Level	Genome Size (Gbp)	Year of Release
*Crotalus horridus*	Timber rattlesnake	ASM162548v1	Contig	1.520	2016
*Crotalus pyrrhus*		CrotMitch1.0	Contig	1.127	2014
*Crotalus tigris*	Tiger rattlesnake	ASM1654583v1	Contig	1.612	2021
*Crotalus viridis viridis*	Prairie rattlesnake	UTA_CroVir_3.0	Chromosome	1.340	2019
*Emydocephalus ijimae*		EmyIji_1.0	Scaffold	1.625	2019
*Hydrophis cyanocinctus*	Asian annulated sea snake	ASM402372v1	Scaffold	1.390	2019
*Hydrophis hardwickii*	Hardwick’s sea snake	ASM402376v1	Scaffold	1.296	2019
*Hydrophis melanocephalus*		hydMel_1.0	Scaffold	1.403	2019
*Laticauda colubrina*	Yellow-lipped sea krait	latCor_2.0	Scaffold	2.039	2020
*Laticauda laticaudata*	Blue-ringed sea krait	latLat_1.0	Scaffold	1.559	2019
*Naja naja*	Indian cobra	Nana_v5	Chromosome	1.769	2019
*Notechis scutatus*	Mainland tiger snake	TS10Xv2-PRI	Scaffold	1.666	2018
*Ophiophagus hannah*	King cobra	OphHan1.0	Scaffold	1.594	2013
*Pantherophis guttatus*		UNIGE_PanGut_3.0	Scaffold	1.707	2020
*Pantherophis obsoletus*	Western rat snake	UNIGE_PanObs_1.0	Scaffold	1.692	2020
*Protobothrops flavoviridis*	Habu	HabAm_1.0	Scaffold	1.413	2018
*Protobothrops mucrosquamatus*	Chinese habu	P.Mucros_1.0	Scaffold	1.674	2016
*Pseudonaja textilis*	Eastern brown snake	EBS10Xv2-PRI	Scaffold	1.590	2018
*Ptyas mucosa*	Dhaman	UNIGE_Pmuc_v1.0	Scaffold	1.721	2020
*Python bivittatus*	Burmese python	Python_molurus_bivittatus-5.0.	Scaffold	1.435	2013
*Thamnophis elegans*	Western terrestrial garter snake	rThaEle1.pri	Chromosome	1.672	2019
*Thamnophis sirtalis*		Thamnophis_sirtalis-6.0	Scaffold	1.425	2015
*Thermophis baileyi*		DSBC_Tbai_1.0	Scaffold	1.748	2018
*Vipera berus berus*	Common viper	Vber.be_1.0	Scaffold	1.532	2014

**Table 2:** Computational tools used for the genomic detection of repeats and to determine transcript levels to study the impact of repeat proportions in snakes and other eukaryotic genomes.

Application	Tool	Repeat Annotation Approach	Features	Input Format	Year	Reference
Repeats annotations and determination of their genomic abundance	RepeatMasker	Homology-based detection nhmmer, cross_match, ABBlast/WUBlast,RMBlast	Identifies all types of interspersed repeats and low-complexity DNA sequences	FASTA assembled contigs or scaffolds	2013	Smit (2020) [123]
	TRF	Stochastic model of tandem repeats by percent identity and frequency of insertions and deletions	Detects tandem repeats in range from 1 to 2000 bp	FASTA	1999	Benson (1999) [242]
	MGEScan	Hmmer	Identifies LTR and non-LTR retroelements	FASTA	2016	Lee et al. (2016) [243]
	LTRdigest	Local alignment and hidden Markov model-based method	Determines the position of potential LTR retrotransposons or ERV insertions	LTR annotation in GFF3 format	2009	Steinbiss et al. (2009) [244]
	RECON	Pairwise-based similarity and BLAST-based mapping	Identifies and classifies de novo repeat sequence families	FASTA	2002	Bao and Eddy (2002) [245]
	RepeatScout	lmer frequency similarity-based mapping with consensus	Identifies novel repeat families	FASTA	2005	Price et al. (2005) [246]
	RepeatModeler2	A pipeline of multiple algorithm runs (RECON, RepeatScout, and LtrHarvest/Ltr_retriever)	Produces high-quality libraries of TE families and detects LTR structure	FASTA	2020	Flynn et al. (2020) [247]
	RepeatExplorer2	Graph-based reads clustering and characterization of repeats	Assembles repeats and detects novel satellites and TEs	Unassembled short reads in FASTQ	2020	Novák et al. (2020) [248]
	dnaPipeTE	RepeatMasker-based mapping	Quantifies the proportion of TEs in unassembled small datasets	FASTQ reads	2015	Goubert et al. (2015) [249]
	REPdenovo	k-mer counting and de novo assembly of repeats	Generates longer repeats	Paired-end FASTQ reads	2016	Chu et al. (2016) [250]
	RepLong	Constructs a network of read overlaps based on pairwise alignment	Identifies novel repetitive elements in the genome using PacBio long reads	FASTA	2018	Guo et al. (2018) [251]
	RepARK	Abundant *k*-mers-based analysis of NGS reads	Detects repetitive motifs and annotates TE classes	FASTQ reads	2014	Koch et al. (2014) [252]
Repeats expression	RepEnrich	Bowtie2-based sensitive mapping of RNA sequences	Assigns repetitive elements into families and subfamilies and identify transcripts	ChIP-seq and RNA-seq FASTQ reads	2014	Criscione et al. (2014) [253]
	TEtranscripts	Uniq and multimode mapping	Accurate differential expression analysis of repeats	GTF files, RNA-seq alignments BAM files	2015	Jin et al. (2015) [254]
	SalmonTE	k-mer-based quasi-mapping	Fast and scalable quantification of TE transcripts	FASTQ files and phenotype data	2018	Jeong et al. (2018) [255]
	TEcandidates	Bowtie2-based mapping	Differential expression analysis of TEs	FASTQ or FASTA	2018	Valdebenito-Maturana et al. (2020) [256]

## Data Availability

All analyses were performed on previously published and publicly available data: accession numbers are provided as Table S1.

## Supplementary Materials

The following are available online at https://www.mdpi.com/article/10.3390/cells10071707/s1, Supplementary Note S1: A description of the methodology used for the transcriptomic analysis of snake repeats and data presented as Figure 3; Figure S1: Workflow pipeline for determining the expression of snake repeats; Figure S2: Heatmap illustrating differential expression data of genomic repeats in Indian cobra. Comparisons are shown for different tissue samples and repeat families on the x-axis and y-axis, respectively. Table S1: Details of samples used for the transcriptomic analysis of repeat elements.
