# Supplementary material for: Impact of Repetitive DNA Elements on Snake Genome Biology and Evolution
Source: Cells. 2021 Jul 6;10(7):1707. doi: 10.3390/cells10071707 (PMC8303610; doi:10.3390/cells10071707)
Supplement: Supplementary file 1 [file cells-10-01707-s001.zip › cells-1244904-supplementary.pdf]

Table S1. Details of samples used for transcriptomic analysis of repeat elements.

Bioproject ID	Biosample ID	Tissue	Number of sequenced bases	SRA Accession ID
PRJNA527614 Indian Cobra	SAMN11168471	Spleen	9.9 Gb	SRR8754982
	SAMN11168472	Brain	16.7Gb	SRR8754983
	SAMN11168473	Heart	18.5 Gb	SRR8754985
	SAMN11168476	Venom gland	25.8 Gb	SRR8754986
	SAMN11168475	Pancreas	20.5 Gb	SRR8754987
	SAMN11168477	Liver	17.2 Gb	SRR8754989
PRJNA477004 Prairie Rattlesnake	SAMN09463176	Female liver	1.1 Gb	SRR7401978
	SAMN09463178	Female kidney	940 MB	SRR7401980
	SAMN09463180	Male liver	1.1 Gb	SRR7401982
	SAMN09463182	Male kidney	1.2 Gb	SRR7401984
	SAMN07375499	Venom	8 Gb	SRR5858075

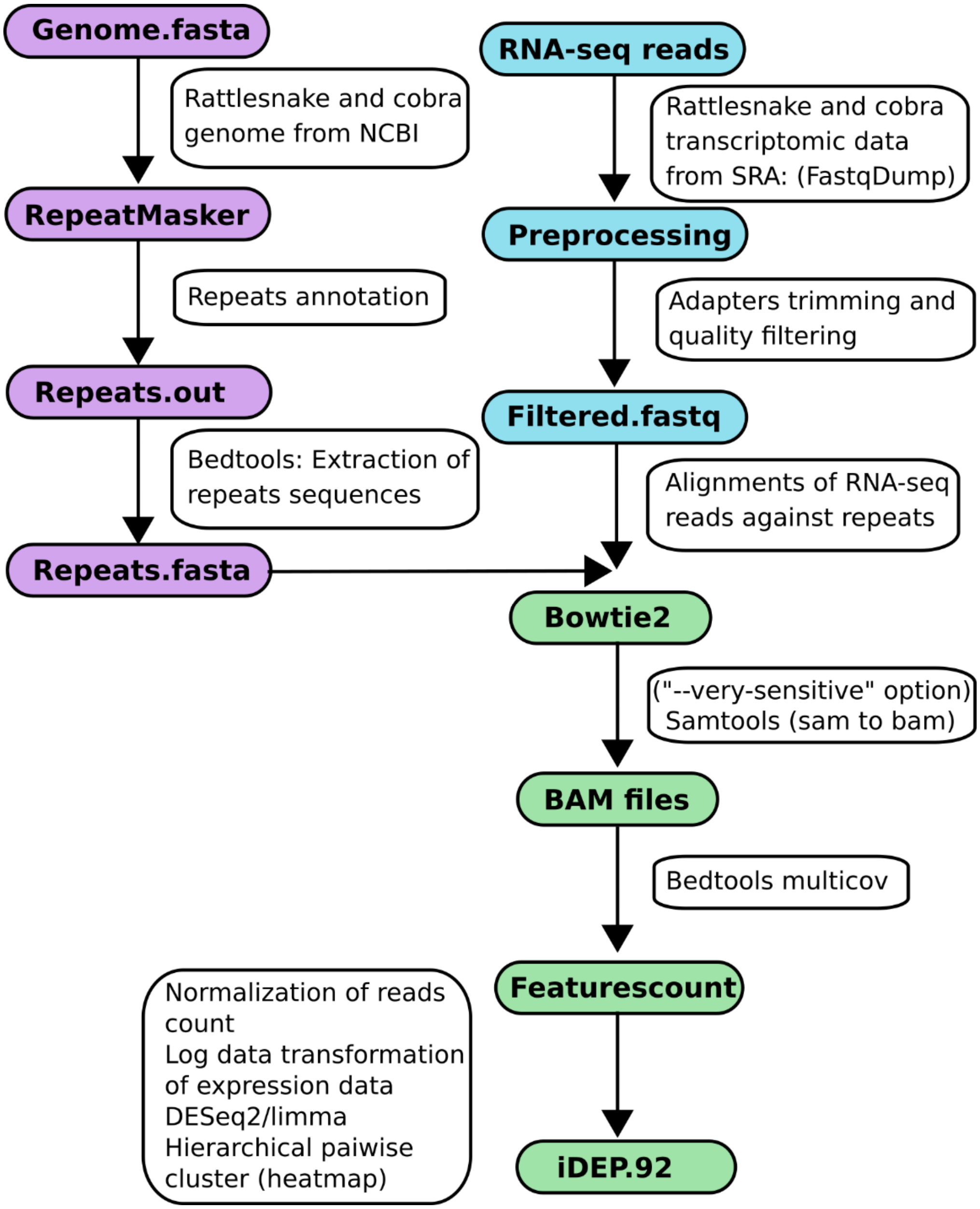

Figure S1. Workflow pipeline for measuring the expression of snake repeats.

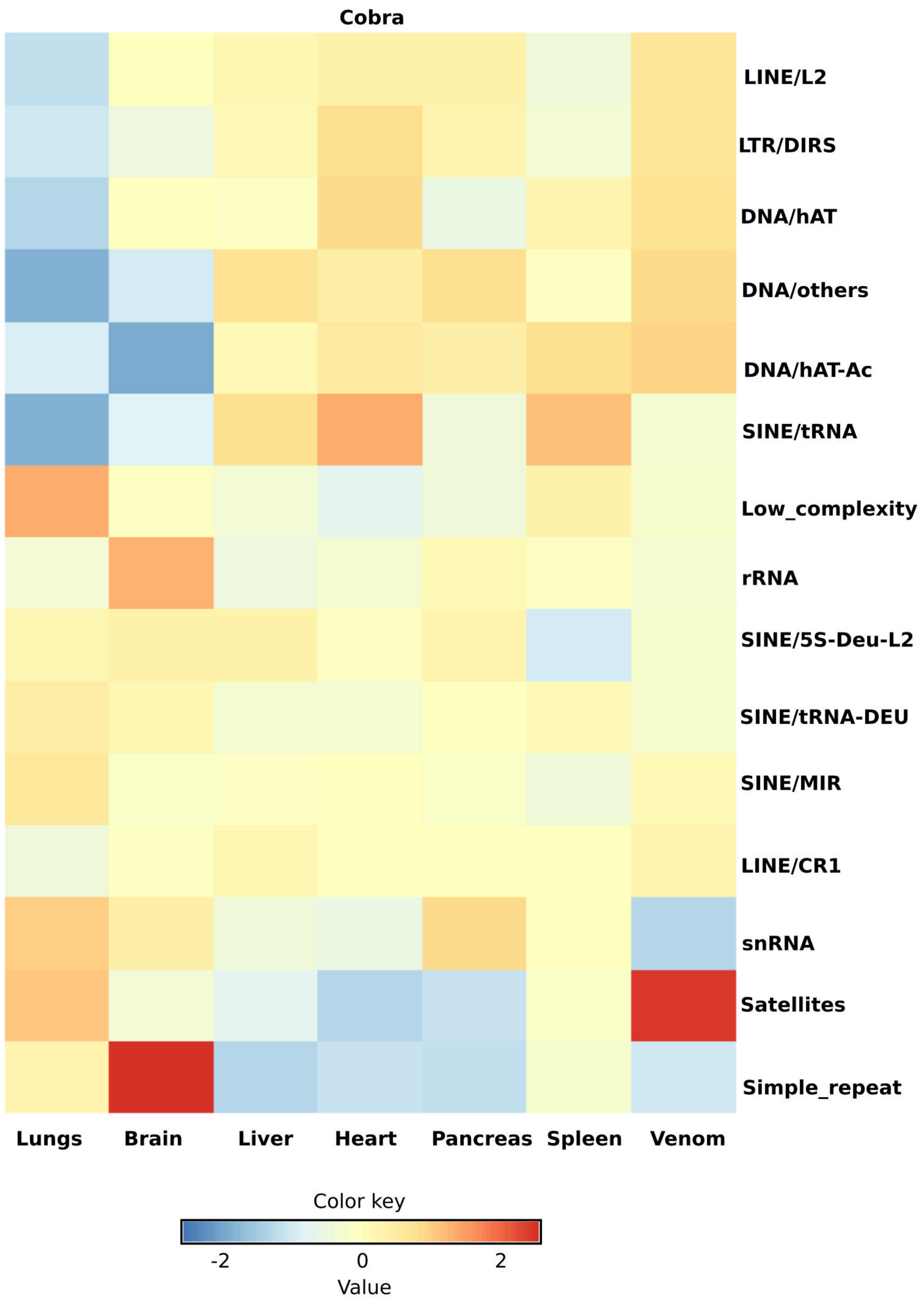

Figure S2. Heatmap illustrating differential expression data of genomic repeats in Indian cobra. Comparisons are shown for different tissue samples and repeat families on the x-axis and y-axis, respectively
